# Spatial distribution and cluster analysis of road traffic accidents in Nepal

**DOI:** 10.1371/journal.pone.0331333

**Published:** 2025-08-29

**Authors:** Roshan Kumar Mahato, Kyaw Min Htike, Alok Kafle, Vishal Gewali, Anup Kafle, Vijay Sharma

**Affiliations:** 1 Faculty of Public Health, Khon Kaen University, Khon Kaen, Thailand; 2 Nepal Public Health and Research Consultancy, Kathmandu Nepal; 3 Tropical Medicine, Faculty of Medicine, Khon Kaen University, Thailand; 4 Kathmandu Valley Traffic Police Office, Kathmandu, Nepal; 5 Arlington County Government, Arlington, Virginia, United States of America; 6 Kathmandu University School of Medical Sciences, Dhulikhel, Nepal; Compass Housing Services Co Ltd. Trading as Home In Place, Newcastle, Australia, AUSTRALIA

## Abstract

**Background:**

Road traffic accidents (RTAs) continue to pose a significant menace to global public health in the form of a high incidence of mortality, disability and economic expense. Their space-time trends are of importance for policy decision-making. This particular study employed spatial analysis to identify high-risk zones and found significant clustering of accidents in urban centers as well as increasing semi-urban and rural vulnerabilities, supporting the need for safety interventions and road infrastructure improvements in Nepal. This paper aims to determine and analyze the incidence of RTAs in Nepal from 2019 to 2022, primarily focusing on vehicle-types and spatial distribution.

**Methods:**

Data from all seven provinces and Kathmandu Valley Traffic Police Office were analyzed to examine RTAs patterns across 77 districts of Nepal. The data were processed and visualized using Quantum GIS (QGIS), and spatial analysis performed using Global and Local Moran’s I statistics, along with Local Indicators of Spatial Association (LISA), to identify spatial clusters of accidents.

**Results:**

This study identified statistically significant spatial clustering of vehicle types involved in RTAs. High-High (HH) clusters, indicating areas with elevated accident rates surrounded by similarly high-risk zones were concentrated in urban centers particularly Kathmandu, Lalitpur, and Bhaktapur. Conversely, Low-Low (LL) clusters, reflecting lower accident rates in sparsely populated regions, were observed in rural areas. Temporal analysis revealed a steady rise in RTAs incidence, with rates increasing from 63.35 per 100,000 population in 2019–2020 (Moran’s *I* = 0.741) to 94.46 in 2020–2021 (Moran’s *I* = 0.595) and 123.05 in 2021–2022 (Moran’s *I* = 0.556).

**Conclusion:**

This present study observed the growing incidence of RTAs in Nepal. The results highlight the critical need for geographically tailored road safety interventions with priority given to urban and semi-urban zones. Effective strategies should emphasize enhanced road traffic law enforcement, strict regulation of commercial and two-wheeled vehicles as well as targeted infrastructure upgrades in an effective manner.

## Introduction

Road traffic accidents (RTAs), defined as collisions and consist of moving vehicles on public or accessible private roads resulting in injuries or fatalities [1]. These remain a significant global challenge. Between 2010 and 2021, global road traffic fatalities decreased by 5% even though concurrent growth was present in population, vehicle use, and infrastructure development [2]. This decline represents to a 16% reduction in deaths per 100,000 population and a 41% decrease per 100,000 vehicles, indicating progress in safety measures but cyclist fatalities have risen, potentially connected to escalating e-bike usage [3]. The economic impact is considerable, with RTAs estimated to cost the global economy $1.8 trillion between 2015 and 2030, with low- and middle-income countries facing as the greatest health burden [4].

In Nepal, motorcycle accidents account for 68% of road traffic accident related fatalities and injuries while bicycles, buses and pedestrian incidents made up smaller portions [5]. Primary contributing factors include driver negligence, speeding and driving under the influence of alcohol drinking [6]. A 2022 Kathmandu study supported these findings that the primary cause of road traffic crashes were driver negligence, excessive speed and alcohol consumption [7]. Less frequent accidents include pedestrian error, unfavourable weather conditions, and junction-related factors [8]. Study from Dharan, Koshi Province of Nepal in 2014 found that heavy vehicles are often involved in fatal pedestrian accidents.

Analysis of Nepal’s road safety landscape reveals four critical themes. These themes are Blame allocation, safety culture, infrastructure quality and user behaviour [9–11]. Blame allocation looks at how the charge for an accident is shared among the parties involved which may have implications for responsibility, policy and governance. Safety culture is a reflection of the societal and institutional perception of road safety [12]. Infrastructure includes road conditions, signposts, lighting and traffic control systems which bear a direct impact on accident risk [13]. User behaviors also comprise actions of drivers (speeding, drinking, use of safety devices and compliance) and actions of pedestrians [14].

Even with the available literature on the causes of road traffic accidents in Nepal, the analysis of the geographic and vehicular patterns remains sparse [15]. Prior spatial analyses have centered on the Kathmandu Valley or certain highways and lack a more comprehensive national scope [16]. Using spatial analysis in road safety study is beneficial because it identifies and spatially analyzes the concentration of accidents and the distribution of vehicles involved in the accidents [17–19]. This means, in turn, that these tailored strategies can be more successfully applied, proven infrastructure is available, appropriate legal control can be enacted and safety outcomes can be improved through predictive modeling [17,18,20]. This paper aims to determine and analyze the incidence of RTAs in Nepal from 2019 to 2022, primarily focusing on vehicle-types and spatial distribution.

## Materials and methods

### Study area

This study was conducted in Nepal covering 77 districts. There are seven provinces alongside three ecological regions which encompass both urban and rural areas [19]. The study uncovered specific geographic areas for the concentration of road traffic accidents. In the densely populated Terai region, the rate of RTAs was very high because the region was traffic congested, the roads were not appropriately developed, and the rate of vehicle traffic was very high [21]. On the other hand, Himalayan districts (elevation 2500–8800 meters above sea level) were facing higher accident risks because of difficult mountainous roads, exposed to the risk of very slow narrow roads, and sharp narrow turning roads [22]. Most urban areas, the Kathmandu Valley in particular, are a disproportionate contributor to the RTAs of the country, which caused a high number of RTAs probably because of the high concentration of motorcycles and small cars, bad weather, and often disregarded traffic rules [23].

### Source of data

This study used the official road traffic accident reports gathered from all seven provincial traffic police headquarters of Nepal (Koshi Province – Itahari, Madhesh Province – Pathlaiya, Bagmati Province – Ramnagar Chitwan, Gandaki Province – Kaski, Lumbini Province – Butwal, Karnali Province – Surkhet, and Sudurpaschim Province – Kailali) and the Kathmandu Valley Traffic Police Office, both organizations under the umbrella of the Ministry of Home Affairs [24]. Data were gathered from January to April of 2024 for three budget years (2019–2020–2021–2022) to have complete temporal representation. The dataset included major variables such as vehicle types involved, severity of accidents (categorized as fatalities, serious injury, and slight injury), and geographical location. The raw data were pre-processed for analysis by: (1) removal of duplicate records, (2) standardization and correction of mis-classification errors in vehicle types, (3) cross-checking to correct missing or incomplete records and (4) systematic verification to determine accuracy and consistency of all the provincial records. This thorough process provided a robust dataset for the subsequent spatial and statistical analyses to be performed.

### Definition and coding of study variables

#### Independent variables.

The analysis was based on five vehicle-type independent variables that defined the annual number of road traffic accidents involving: (1) trucks, (2) tippers (heavy construction material carriers), (3) buses (public and private), (4) cars (commercial and private), and (5) motorbikes. These quantitative continuous variables were coded as follows: Truck RTAs (RTAs involving trucks), Tipper RTAs (RTAs involving tippers), Bus RTAs (RTAs involving buses), Car RTAs (RTAs involving cars) and Motorbike RTAs (RTAs involving motorbikes). This coding facilitated uniformity among the records and focused the assessment to the vehicle accident patterns.

#### Outcome variables.

For this specific study, the defining outcome variable was the occurrence of Road Traffic Accidents. The fiscal year 15th July 2019–15th June 2020 is the period during which the newly recorded road traffic accident cases were counted. In this case, the numerator is the newly reported RTAs and the population remains constant during the year yielding a rate of 100,000 population [25].

### Statistical analysis

Once the dataset was validated, the data was imported into Quantum Geographic Information System version 3.36 (Maidenhead) to combine spatial and non-spatial data by creating a shapefile for this analysis [26]. Using QGIS, we visualized the severity rates of RTAs from FY 2019–2022 identifying the spatial distribution of road traffic accidents across Nepal. Following this, a detailed spatial analysis was carried out using GeoDa version 1.22 [27].

### Univariate and multivariate risk factor analysis

The analysis was begun by applying Global and Local Moran’s I statistics to explore spatial autocorrelation in RTAs severity rates. Global Moran’s I was used to detect overall spatial patterns across the country while Local Moran’s I was employed to identify localized clusters or hotspots [28]. This study was used 999 permutations to evaluate how the significance of locations varied with the number of permutations while maintaining a significance threshold of p < 0.05. The Global Moran’s I was calculated using the following formula,


$$I=\frac{n}{S_{\text{0}}}*\frac{Z_{i}Z_{j}W_{ij}}{Z_{i}^{\text{2}}}\text{\textbackslash{} }$$ (Eq.1)

Local Moran’s I was computed as:


$$I_{1}=\frac{Z_{i}}{S_{\text{1}}}*Z_{j}W_{ij}$$ (Eq.2)

Where ‘n’ is the total number of regions, S_0_’ represents the sum of all spatial weights, Z’ is the deviation of the variable from its mean, and ‘S_1_’ indicates the sum of all squared deviations. ‘W_ij_’ refers to the spatial weight between regions i and j.

In this case, Global Moran’s I and Local Indicators of Spatial Autocorrelation were used for assessing global and local RTAs severity rates and vehicle types spatial autocorrelation. More specifically, Moran’s I within LISA was used to explore the question of whether the given regions with RTAs severity rates clustered spatially (high-high or low-low clusters) or were spatial outliers (high-low or low-high or vice versa).

While looking at the data for LISA, High-High clusters are geographical areas exhibiting high RTA severity rates along with their high neighboring rates, thus marking them as hotspots. Low-Low clusters are the areas with low RTA severity rates together with their neighboring areas also exhibiting low RTA rates, thus marking them as cold spots. High-Low (HL) clusters are areas with high RTA severity rates along with their neighboring areas with low rates which marks them as outliers in the region. Low-High (LH) clusters consist of areas with low RTA rates bordering areas with high RTA rates, thus also marking them as outliers.

To determine these spatial relationships, we used a “K-nearest neighbors” (KNN) approach with its three closest neighboring districts. These nearby neighbors are identified based on geographic distance. Both univariate LISA (analyzing RTAs severity alone) and bivariate LISA (analyzing RTAs severity in relation to various vehicle types) were applied in this study to identify significant spatial clusters across Nepal.

### Ethical consideration

This study (HE672162) received an exemption from ethics review by the Khon Kaen University Ethics Committee for Human Research (KKUEC) on August 20, 2024. In addition, this study also collected and approval letter from the Kathmandu Valley Traffic Police Office. This ethical exemption was granted because of the permission letter and the study also involved secondary data analysis with identifiable information which did not require explicit consent. All required permissions for accessing the data were secured.

## Results

### Incidence of road traffic accident in Nepal

In the fiscal year 2019–2020, the rate of RTAs along with the 100,000 population also had 63.35, with a Moran’s I of 0.741 and Z score of 10.04 which marks spatial significance. HH clusters where RTAs rates were high and observed in the districts of Kathmandu, Lalitpur, Bhaktapur, Makawanpur, Dhading, Rasuwa, Sindhupalchok, Kabhrepalanchok, and Nuwakot. LL clusters where RTAs were low in Kaski, Humla, Bajhang, Bajura, Doti, Jumla and Rukum West districts.

The fiscal year of 2020–2021 witnessed an increase in RTAs along with the population which increased to 94.46 for every 100,000 population, with a Moran’s I of 0.595 and a z score of 7.53 which still demonstrates spatial significance. HH clusters remained constant and were found in Kathmandu, Lalitpur, Bhaktapur and also found in new districts of Sunsari, Makawanpur, Sarlahi and Sitara. The emergence of the LL clusters has been observed in the districts of Parbat, Gulmi, Rukum East, Surkhet, Kalikot, Jumla, Bajura, Bajhang and Humla.

**Fig 1:**
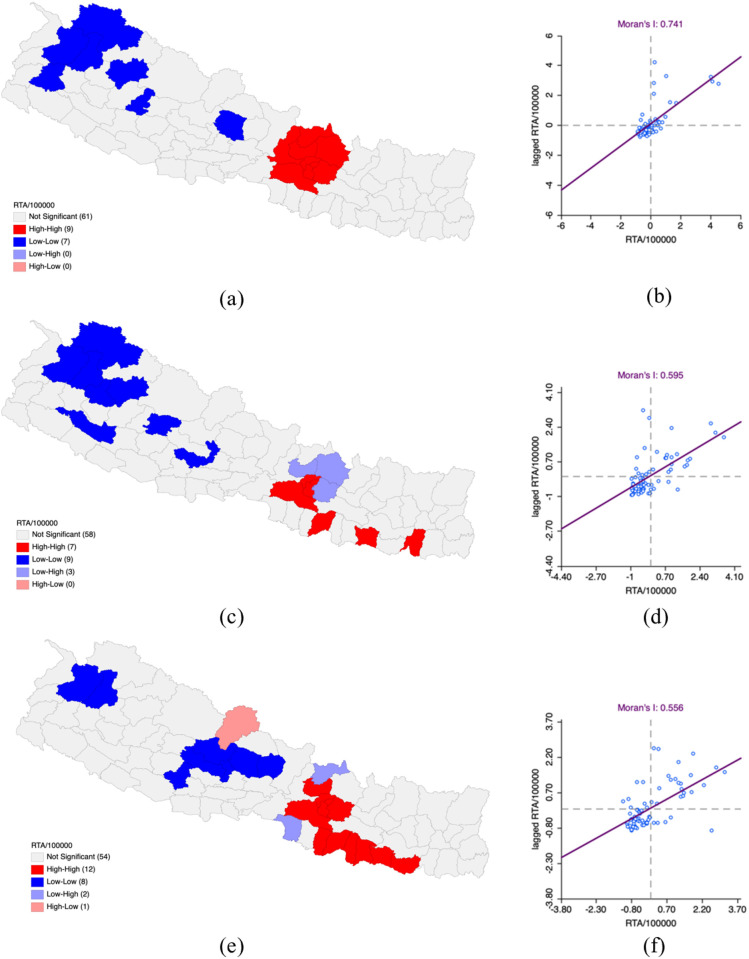
Incidence of Road Traffic Accident per 100,000 population in Nepal FY 2019–2022. (a) LISA map of Road Traffic Accident per 100,000 population in FY 2019–2020. (b) Moran’s I scatter plot of Road Traffic Accident per 100,000 population in FY 2019–2020. (c) LISA map of Road Traffic Accident per 100,000 population in FY 2020–2021. (d) Moran’s I scatter plot of Road Traffic Accident per 100,000 population in FY 2020–2021. (e) LISA map of Road Traffic Accident per 100,000 population in FY 2021–2022. (f) Moran’s I scatter plot of Road Traffic Accident per 100,000 population in FY 2021–2022. “Republished from Registration Number 155765 under a CC BY license, with permission from Hermes Engineering Solution, original copyright 2021”.

As of FY 2021–2022, the incidence of RTAs has increased to 123.05 per 100,000 population with a Moran’s I value of 0.556 and a z score of 7.16, still showing spatial significance. HH clusters were noted in the Kathmandu, Lalitpur, Bhaktapur, Makawanpur, Sarlahi, Dhanusha, Saptari, Nuwakot, Kabhrepalanchok, Rautahat, Mahottari and Siraha districts. LL clusters were identified in Bajhang, Bajura, Pyuthan, Baglung, Parbat, Myagdi, Kaski, and Lamjung districts (Table 1 and Fig 1).

**Table 1:** Incidence of RTAs per 100,000 population in Nepal FY 2019–2022.

Fiscal Year	LISA				Moran’s I
	HH	LL	HL	LH	
**2019**–**2020**	Kathmandu*** Lalitpur*** Bhaktapur*** Makawanpur*** Dhading* Rasuwa** Sindhupalchok* Kabhrepalanchok*** Nuwakot***	Kaski* Humla* Bajhang*** Bajura* Doti* Jumla** Rukum West*			0.741
**2020**–**2021**	Kathmandu** Lalitpur*** Bhaktapur** Sunsari* Makawanpur*** Sarlahi* Siraha*	Parbat* Gulmi* Rukum East* Surkhet* Kalikot** Jumla** Bajura** Bajhang*** Humla**		Sindhupalchok* Kabhrepalanchok*** Nuwakot***	0.595
**2021**–**2022**	Kathmandu** Lalitpur*** Bhaktapur** Makawanpur** Sarlahi* Dhanusha* Saptari* Nuwakot*** Kabhrepalanchok*** Rautahat* Mahottari* Siraha*	Bajhang* Bajura* Pyuthan* Baglung* Parbat* Myagdi* Kaski* Lamjung**	Mustang**	Rasuwa* Parsa*	0.556

P-value *** 0.001, ** 0.01, *0.05

### Situation of truck induced road traffic accidents in Nepal

In FY 2019–2020, the bivariate LISA analysis showed positive spatial autocorrelation with truck density and the number of road traffic accidents (RTAs) per 100,000 population with a Moran’s I value of 0.553. Hotspot clusters were identified in and around Kathmandu, Lalitpur, Bhaktapur, and Makawanpur and Dhading districts, which not only had high truck density and RTAs but were also surrounded by districts with comparable high values. Coldspot clusters were identified in Kaski, Humla, Bajhang, Bajura, Doti, Jumla and Rukum West, where both truck density and RTAs were low and were surrounded by areas with low values.

For FY 2020–2021, the positive spatial autocorrelation diminished to slightly 0.394 (Moran’s I) compared to FY 2019–2020. Hotspot areas still remained in Kathmandu, Lalitpur, Bhaktapur and Makawanpur and newly added were Sunsari, which had both high truck density and RTAs. Coldspot clusters were identified in Parbat, Gulmi, Rukum East, Surkhet, Kalikot, Humla, Doti, Bajura, Jumla and Bajhang where both variables were low and were surrounded by areas with comparable low values.

**Fig 2:**
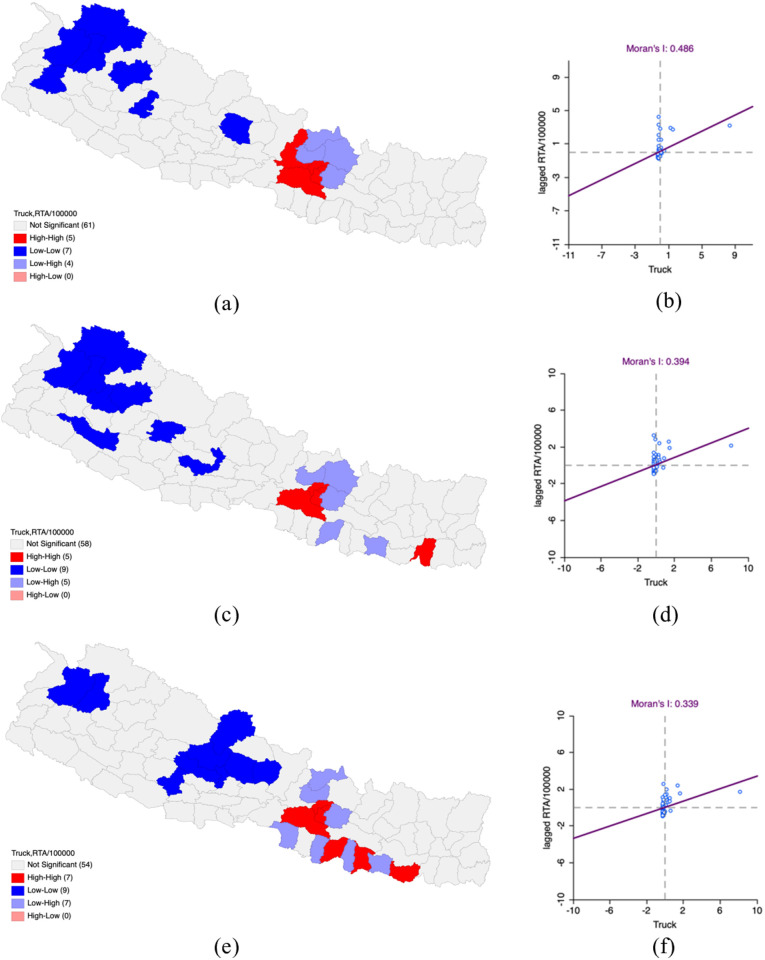
Impact of truck on Road Traffic Accidents per 100,000 population in Nepal FY 2019–2022. (a) LISA map of Truck with RTA per 100,000 population in FY 2019–2020. (b) Moran’s I scatter plot of Truck with RTA per 100,000 population in FY 2019–2020. (c) LISA map of Truck with RTA per 100,000 population in FY 2020–2021. (d) Moran’s I scatter plot of Truck with RTA per 100,000 population in FY 2020–2021. (e) LISA map of Truck with RTA per 100,000 population in FY 2021–2022. (f) Moran’s I scatter plot of Truck with RTA per 100,000 population in FY 2021–2022. “Republished from Registration Number 155765 under a CC BY license, with permission from Hermes Engineering Solution, original copyright 2021”.

In the deductions of Bivariate LISA and in the analysis, it can be observed that the previous fiscal year of 2020−2021 has a slight decrease of the value in spatial correlation with a total positive correlation of Moran’s I-0.339. Hotspot clusters are present in the remaining districts of Kathmandu, Lalitpur, Bhaktapur, Makawanpur, Sarlahi, Dhanusha and Saptari indicating higher values of RTAs in relation to the level of trucks in the surrounding districts. Bajhang, Bajura, Pyuthan, Baglung, Parbat, Myagdi, Mustang, Kaski and Lamjung were in the coldspot clusters, stating that the remaining districts represent lower value RTAs in relation to the truck density in those districts (Table 2 and Fig 2).

**Table 2:** Impact of truck on Road Traffic Accidents per 100,000 population in Nepal FY 2019–2022.

Fiscal Year	LISA				Moran’s I
	HH	LL	HL	LH	
**2019–2020**	Kathmandu*** Lalitpur*** Bhaktapur*** Makawanpur*** Dhading*	Kaski* Humla* Bajhang*** Bajura* Doti* Jumla** Rukum West*		Sindhupalchok* Rasuwa** Kabhrepalanchok*** Nuwakot***	0.486
**2020–2021**	Kathmandu** Lalitpur*** Bhaktapur** Sunsari* Makawanpur***	Parbat* Gulmi* Rukum East* Surkhet* Kalikot** Jumla** Bajura** Bajhang*** Humla**		Sindhupalchok* Kabhrepalanchok*** Sarlahi* Siraha* Nuwakot***	0.394
**2021–2022**	Kathmandu** Lalitpur*** Bhaktapur** Makawanpur** Sarlahi* Dhanusha* Saptari*	Bajhang* Bajura* Pyuthan* Baglung* Parbat* Myagdi* Mustang** Kaski* Lamjung**		Rasuwa* Nuwakot*** Kabhrepalanchok*** Parsa* Rautahat* Mahottari* Siraha*	0.339

P-value *** 0.001, ** 0.01, *0.05

### Situation of Tipper on Road Traffic Accidents in Nepal

In FY 2019−2020, the results from bivariate LISA analysis revealed a positive spatial correlation between the density of tippers and the number of road traffic accidents (RTAs) per 100,000 population (Moran’s I – 0.553). The High-High clusters included the districts of Kathmandu, Lalitpur, Bhaktapur, Kabhrepalanchok, and Nuwakot, which both tipper density and RTA rates were high. Moreover, these districts were adjacent to other districts which also had high values. Low-Low clusters were detected in Kaski, Humla, Bajhang, Bajura, Doti, Jumla, and Rukum West where both tipper density and RTA rates were low.

By FY 2020−2021, there was a slight decrease in positive spatial correlation compared to FY 2019−2020 (Moran’s I – 0.464). High-high clusters included the previously mentioned districts of Kathmandu, Lalitpur, Bhaktapur, and new ones like Sunsari Nuwot and Makawanpur which had high tipper density and RTAs. New Low-Low clusters included Parbat, Gulmi, Rukum East, Surkhet, Kalikot, Jumla, Bajura, Bajhang, and Humla where both tipper density and RTAs were low.

**Fig 3:**
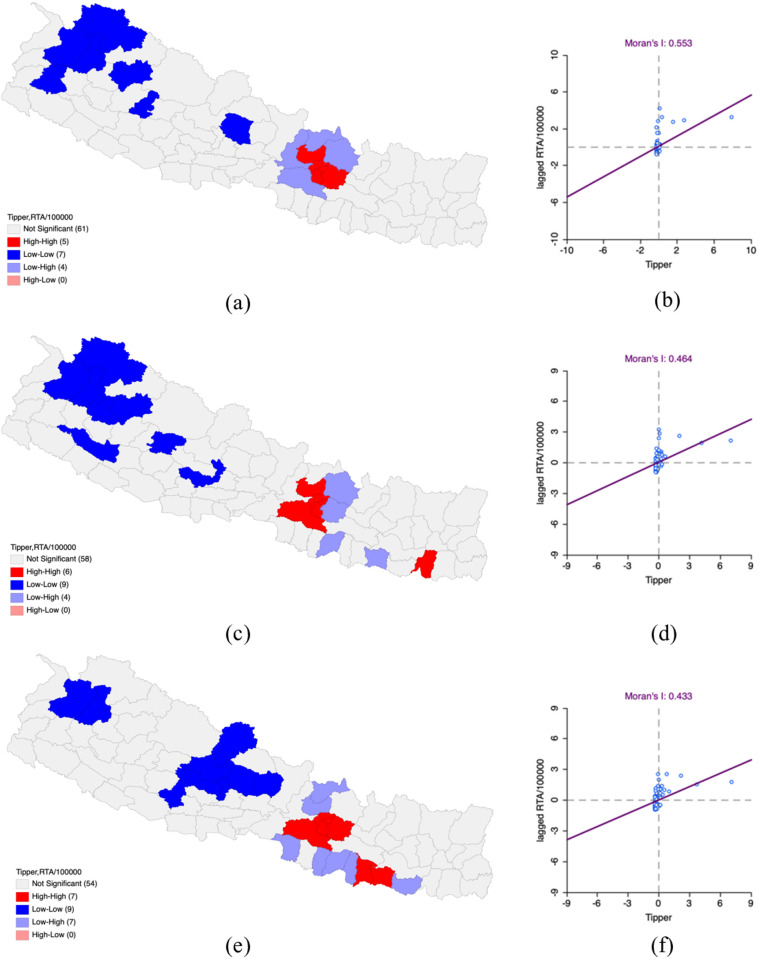
Impact of tipper on Road Traffic Accidents per 100,000 population in Nepal FY 2019–2022. (a) LISA map of Tipper with RTA per 100,000 population in FY 2019–2020. (b) Moran’s I scatter plot of Tipper with RTA per 100,000 population in FY 2019–2020. (c) LISA map of Tipper with RTA per 100,000 population in FY 2020–2021. (d) Moran’s I scatter plot of Tipper with RTA per 100,000 population in FY 2020–2021. (e) LISA map of Tipper with RTA per 100,000 population in FY 2021–2022. (f) Moran’s I scatter plot of Tipper with RTA per 100,000 population in FY 2021–2022. “Republished from Registration Number 155765 under a CC BY license, with permission from Hermes Engineering Solution, original copyright 2021”.

With spatially positive correlation Moran’s I – 0.339 in FY 2021–2022 bivariate LISA analysis showed that RTAs were slightly declining in spatially positive correlation in comparison to FY 2020–2021. High-High clusters were constructed as a result of supplementary concentrations RTAs and identified in districts Kathmandu, Lalitpur, Bhaktapur, Makawanpur, Siraha and Dhanusha and Saptari. Low-Low clusters were noted in districts Bajhang, Bajura, Pyuthan, Baglung, Parbat, Myagdi, Mustang, Kaski and Lamjung where both tipper density and RTAs were low along with neighboring districts as well (Table 3 and Fig 3).

**Table 3:** Impact of tipper on Road Traffic Accidents per 100,000 population in Nepal FY 2019–2022.

Fiscal Year	LISA				Moran’s I
	HH	LL	HL	LH	
2019–2020	Kathmandu*** Lalitpur*** Bhaktapur*** Kabhrepala nchok*** Nuwakot***	Kaski* Humla* Bajhang*** Bajura* Doti* Jumla** Rukum West*		Sindhupalchok* Makawanpur*** Rasuwa** Dhading*	0.553
2020–2021	Kathmandu** Lalitpur*** Bhaktapur** Sunsari* Makawanpur*** Nuwakot***	Parbat* Gulmi* Rukum East* Surkhet* Kalikot** Jumla** Bajura** Bajhang*** Humla**		Sindhupalchok* Kabhrepalanchok*** Sarlahi* Siraha*	0.464
2021–2022	Kathmandu** Lalitpur*** Bhaktapur** Makawanpur** Dhanusha* Saptari* Siraha*	Bajhang* Bajura* Pyuthan* Baglung* Parbat* Myagdi* Mustang** Kaski* Lamjung**		Rasuwa* Nuwakot*** Kabhrepalanchok*** Parsa* Rautahat* Mahottari* Sarlahi*	0.433

P-value *** 0.001, ** 0.01, *0.05

### Situation of bus on road traffic accidents in Nepal

In FY 2019−2020, the bivariate LISA analysis showed that there was a spatially positive correlation between bus density and RTAs per 100,000 population (Moran’s I – 0.440). Bus density and RTAs hotspot clusters were high in Kathmandu, Lalitpur and Bhaktapur districts and neighboring districts. These are bus RTAs coldspot clusters. Kaski, Humla, Bajhang, Bajura, Doti, Jumla and Rukum West were all identified as districts where bus density and RTAs were low, surrounded by areas with low values.

In this fiscal year, 2020–2021, there was a moderately decreased positive spatial correlation (Moran’s I – 0.328) in comparison to FY 2019–2020. The Hotspot clusters where bus density and RTAs were high continued to expand in Kathmandu, Lalitpur and Bhaktapur, adding Sunsari and Makawanpur to the constellation. These were surrounded by districts with similarly high values. Coldspot clusters in Parbat, Gulmi, Rukum East, Surkhet, Kalikot, Jumla, Bajura, Bajhang and Humla showed low RTAs and bus density surrounded by districts exhibiting this pattern.

**Fig 4:**
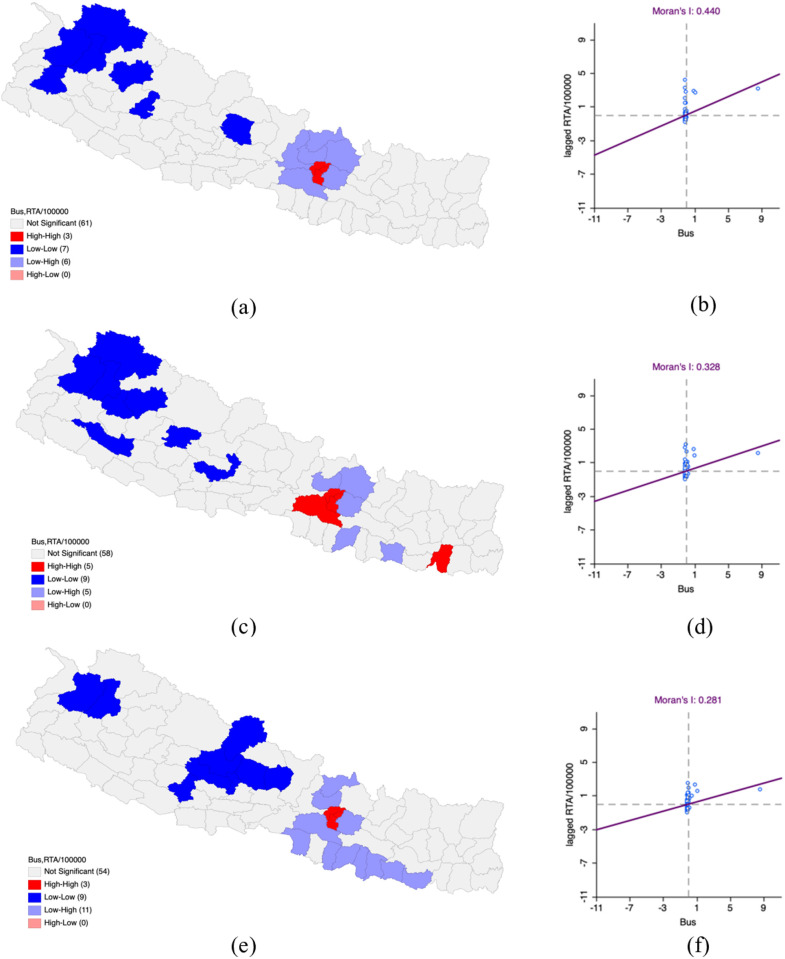
Impact of bus on Road Traffic Accidents per 100,000 population in Nepal FY 2019–2022. (a) LISA map of Bus with RTA per 100,000 population in FY 2019–2020. (b) Moran’s I scatter plot of Bus with RTA per 100,000 population in FY 2019–2020. (c) LISA map of Bus with RTA per 100,000 population in FY 2020–2021. (d) Moran’s I scatter plot of Bus with RTA per 100,000 population in FY 2020–2021. (e) LISA map of Bus with RTA per 100,000 population in FY 2021–2022. (f) Moran’s I scatter plot of Bus with RTA per 100,000 population in FY 2021–2022. “Republished from Registration Number 155765 under a CC BY license, with permission from Hermes Engineering Solution, original copyright 2021”.

In FY 2021–2022, the bivariate LISA analysis indicated persistent decline along with spatially positive correlation (Moran’s I – 0.281) relative to FY 2020–2021. Hotspot clusters were located in the Kathmandu, Lalitpur and Bhaktapur districts which showed high bus ridership and road traffic accident (RTAs) rates with adjacent districts exhibiting the same trend. The coldspot clusters in Bajhang, Bajura, Pyuthan, Baglung, Parbat, Myagdi, Mustang, Kaski and Lamjung districts demonstrated low bus ridership and RTAs, along with similar neighboring districts (Table 4 and Fig 4).

**Table 4:** Impact of bus on Road Traffic Accidents per 100,000 population in Nepal FY 2019–2022.

Fiscal Year	LISA				Moran’s I
	HH	LL	HL	LH	
**2019**–**2020**	Kathmandu*** Lalitpur*** Bhaktapur***	Kaski* Humla* Bajhang*** Bajura* Doti* Jumla** Rukum West*		Nuwakot*** Sindhupalchok* Kabhrepalanchok*** Makawanpur*** Rasuwa** Dhading*	0.440
**2020**–**2021**	Kathmandu** Lalitpur*** Bhaktapur** Makawanpur*** Sunsari*	Parbat* Gulmi* Rukum East* Surkhet* Kalikot** Jumla** Bajura** Bajhang*** Humla**		Nuwakot*** Sindhupalchok* Kabhrepalan chok*** Sarlahi* Siraha*	0.328
**2021**–**2022**	Kathmandu** Lalitpur*** Bhaktapur**	Bajhang* Bajura* Pyuthan* Baglung* Parbat* Myagdi* Mustang** Kaski* Lamjung**		Rasuwa* Nuwakot*** Kabhrepalanchok*** Parsa* Rautahat* Mahottari* Sarlahi* Makawanpur** Dhanusha* Saptari* Siraha*	0.281

P-value *** 0.001, ** 0.01, *0.05

### Situation of Car on Road Traffic Accidents in Nepal

In FY 2019–2020, the bivariate LISA analysis demonstrated spatially positive correlation between car density and RTAs per 100,000 population with Moran’s I value of 0.455. High-High clusters were located in Kathmandu, Lalitpur and Bhaktapur districts which had high car density and RTAs. Low-Low clusters were classified in Kaski, Humla, Bajhang, Bajura, Doti, and Jumla, and Rukum West where low car density and RTAs were prevalent, surrounded by similarly lower regions.

In FY 2020–2021, there was a moderate decrease in the positive spatial correlation (Moran’s I – 0.345) as compared to FY 2020–2021. Hotspot clusters where both car density and RTAs common in Kathmandu, Lalitpur, Bhaktapur and Sunsari, which were surrounded by districts with similarly high values. In contrast, Coldspot clusters were seen in Parbat, Gulmi, Rukum East, Surkhet, Kalikot, Jumla, Bajura, Bajhang and Humla districts where both car density and RTAs were low and surrounded by districts with similar patterns.

**Fig 5:**
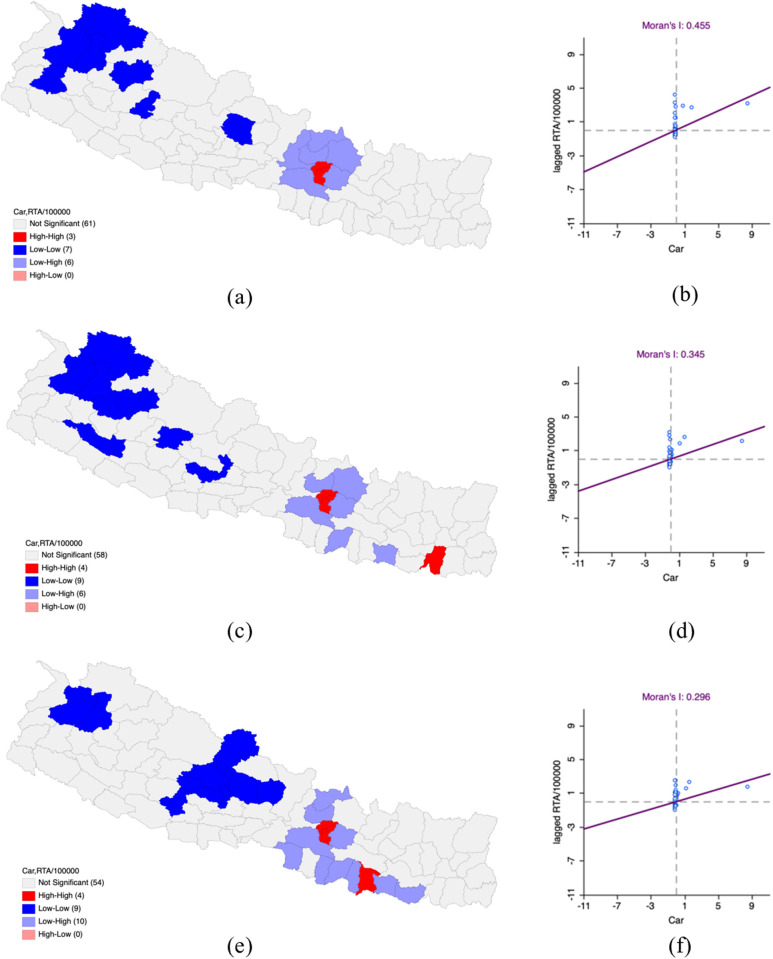
Impact of car on Road Traffic Accidents per 100,000 population in Nepal FY 2019–2022. (a) LISA map of Car with RTA per 100,000 population in FY 2019–2020. (b) Moran’s I scatter plot of Car with RTA per 100,000 population in FY 2019–2020. (c) LISA map of Car with RTA per 100,000 population in FY 2020–2021. (d) Moran’s I scatter plot of Car with RTA per 100,000 population in FY 2020–2021. (e) LISA map of Car with RTA per 100,000 population in FY 2021–2022. (f) Moran’s I scatter plot of Car with RTA per 100,000 population in FY 2021–2022. “Republished from Registration Number 155765 under a CC BY license, with permission from Hermes Engineering Solution, original copyright 2021”.

As of FY 2021–2022, the bivariate LISA analysis revealed that the spatially positive correlation was continued to decline (Moran’s I value – 0.296) in comparison to FY 2020–2021. High-High clusters were noted in Kathmandu, Lalitpur, Dhanusha and Bhaktapur districts, where high car density and RTAs were coupled with neighboring districts having high values as well. At the same time, Low-Low clusters were recorded in Bajhang, Bajura, Pyuthan, Baglung, Parbat, Myagdi, Mustang, Kaski and Lamjung, where both car density and RTAs were low along with surrounding districts exhibiting similarly low values (Table 5 and Fig 5).

**Table 5:** Impact of car on Road Traffic Accidents per 100,000 population in Nepal FY 2019–2022.

Fiscal Year	LISA				Moran’s I
	HH	LL	HL	LH	
2019–2020	Kathmandu*** Lalitpur*** Bhaktapur***	Kaski* Humla* Bajhang*** Bajura* Doti* Jumla** Rukum West*		Nuwakot*** Sindhupalchok* Kabhrepalanchok*** Makawanpur*** Rasuwa** Dhading*	0.455
2020–2021	Kathmandu** Lalitpur*** Bhaktapur** Sunsari*	Parbat* Gulmi* Rukum East* Surkhet* Kalikot** Jumla** Bajura** Bajhang*** Humla**		Nuwakot*** Sindhupalchok* Kabhrepalanchok*** Sarlahi* Siraha* Makawanpur***	0.345
2021–2022	Kathmandu** Lalitpur*** Bhaktapur** Dhanusha*	Bajhang* Bajura* Pyuthan* Baglung* Parbat* Myagdi* Mustang** Kaski* Lamjung**		Rasuwa* Nuwakot*** Kabhrepalanchok*** Parsa* Rautahat* Mahottari* Sarlahi* Makawanpur** Saptari* Siraha*	0.296

P-value *** 0.001, ** 0.01, *0.05

### Situation of Motorbike on Road Traffic Accidents in Nepal

Bivariate LISA analysis revealed a spatially positive correlation between motorbike density and RTAs per 100,000 population, showing a Moran’s I of 0.485 for FY 2019–2020. High-High clusters where both motorbike density and RTAs were elevated, included and were situated in Kathmandu, Lalitpur and Bhaktapur districts which were also bordered by other districts with comparably high values. Low-Low clusters were found Kaski, Humla, Bajhang, Bajura, Doti, Jumla, and Rukum West where both motorbike density and RTAs were low and were surrounded by areas with low values.

By FY 2020–2021, the correlation was slightly decreasing spatially positive (Moran’s I – 0.423) in comparison to FY 2019–2020. High-High clusters where both motorbike density and RTAs were high also included and were identified in Kathmandu, Lalitpur and Bhaktapur and later also included Sunsari, Sarlahi and Siraha which are neighboring districts exhibiting similarly high values. Clusters with low RTAs, like high RTAs, were also consistent. They were found in Gulmi, Rukum East, Surkhet and Kalikot, as well as Bajura, Bajhang and Humla, where both motorbike density and RTAs were low and surrounded by districts equally low.

**Fig 6:**
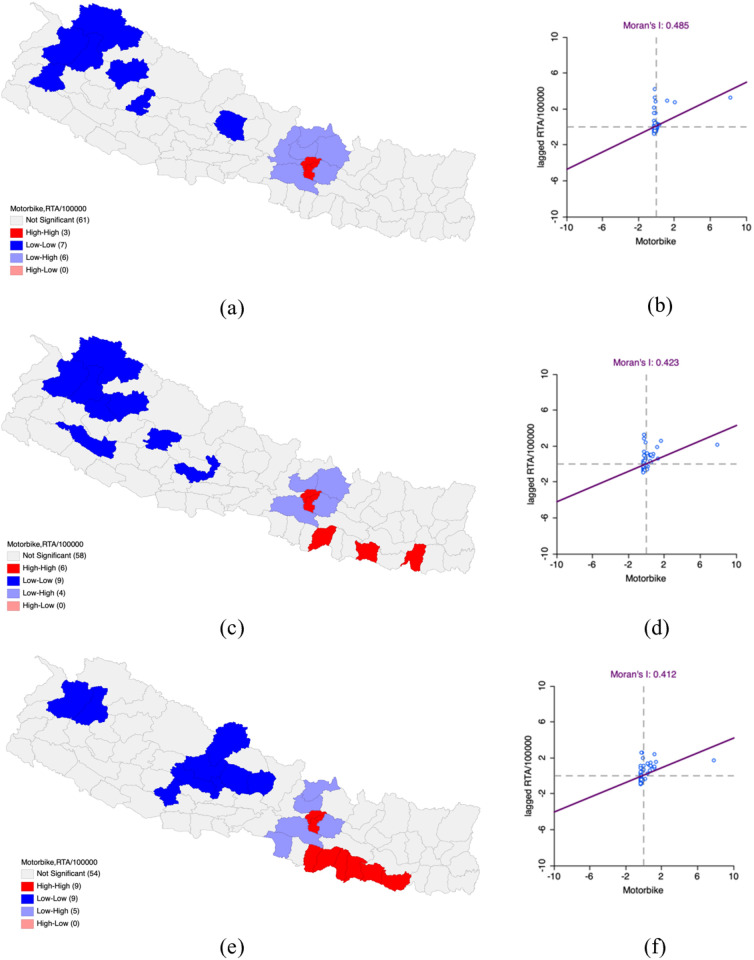
Impact of motorbike on Road Traffic Accidents per 100,000 population in Nepal FY 2019–2022. (a) LISA map of Motorbike with RTA per 100,000 population in FY 2019–2020. (b) Moran’s I scatter plot of Motorbike with RTA per 100,000 population in FY 2019–2020. (c) LISA map of Motorbike with RTA per 100,000 population in FY 2020–2021. (d) Moran’s I scatter plot of Motorbike with RTA per 100,000 population in FY 2020–2021. (e) LISA map of Motorbike with RTA per 100,000 population in FY 2021–2022. (f) Moran’s I scatter plot of Motorbike with RTA per 100,000 population in FY 2021–2022. “Republished from Registration Number 155765 under a CC BY license, with permission from Hermes Engineering Solution, original copyright 2021”.

As compared to the previous FY 2020−2021, FY 2021−2022 showed a decrease in bivariate LISA analysis spatially positive correlation (Moran’s I – 0.412). The identified High-High clusters in the motorbike dense and RTAs dense areas included Kathmandu, Lalitpur, Dhanusha, Sarlahi, Rautahat, Mahottari, Saptari, Siraha and Bhaktapur. In these areas, motorbike dispersion and RTAs were high along with the surrounding districts. In Bajhang, Bajura, Pyuthan, Baglung, Parbat, Myagdi, Mustang, Kaski and Lamjung, Low-Low clusters were identified where motorbike dispersion, RTAs and the RTAs of these districts were low were low reflecting the values of their neighboring districts (Table 6 and Fig 6).

**Table 6:** Impact of motorbike on Road Traffic Accidents per 100,000 population in Nepal FY 2019-2022.

Fiscal Year	LISA				Moran’s I
	HH	LL	HL	LH	
2019–2020	Kathmandu*** Lalitpur*** Bhaktapur***	Kaski* Humla* Bajhang*** Bajura* Doti* Jumla** Rukum West*		Nuwakot*** Sindhupalchok* Kabhrepalanchok*** Makawanpur*** Rasuwa** Dhading*	0.485
2020–2021	Kathmandu** Lalitpur*** Bhaktapur** Sunsari* Sarlahi* Siraha*	Parbat* Gulmi* Rukum East* Surkhet* Kalikot** Jumla** Bajura** Bajhang*** Humla**		Nuwakot*** Sindhupalchok* Kabhrepalanchok*** Makawanpur***	0.423
2021–2022	Kathmandu** Lalitpur*** Bhaktapur** Dhanusha* Sarlahi* Rautahat* Mahottari* Saptari* Siraha*	Bajhang* Bajura* Pyuthan* Baglung* Parbat* Myagdi* Mustang** Kaski* Lamjung**		Rasuwa* Nuwakot*** Kabhrepalanchok*** Parsa* Makawanpur**	0.412

P-value *** 0.001, ** 0.01, *0.05

## Discussion

This paper aims to determine and analyze the incidence of RTAs in Nepal from 2019 to 2022, primarily focusing on vehicle-types and spatial distribution. The results from the Moran’s I and z-scores analysis demonstrated that there is considerable spatial clustering of RTAs which showed that some regions are more affected by RTAs and localized determinants of RTAs are critical. The HH clusters which were present from the years 2019–2022 in areas like Kathmandu, Lalitpur and Bhaktapur showed the relationship between population density, urbanization and motor vehicle traffic in increasing RTAs rates. Our findings were aligned with a study from Nepal and found crash hotspots in tandem with high population density and urbanization in the Valley from 2019 to 2021 [29]. In the same way, another study from Nepal also documented the presence of fatal and major crashes along heavily traveled arterial roadways including the Araniko Highway and significant intersections within the Banepa Municipality, demonstrating the influence of roadway design and vehicle traffic on crashes in urban areas [30]. At the country level, a study from Nepal reported a significant increasing trend in RTAs over a decade span in FY 2009−10 approximately 7,000 incidents were recorded and by FY 2019−20 this figure rose to over 16,000 highlighting the increasing danger of road crashes in the country before the year 2019 [31].

Moreover, the remote LL cluster regions such as Bajura, Bajhang and Humla, recorded low fatality densities. While motorization may be lower in these areas, it may also indicate lower reporting of RTAs in these regions due to limited surveillance and access to healthcare. In relation to healthcare underreporting, a study from Nepal found that police reporting captured only 62.5% of recorded fatalities, 11.6% of property damages and 7.1% of recorded minor injuries and this illustrates the significant underreporting of the healthcare burden [32]. This is due to the persisting cluster concentration in the urban areas fueled by the higher vehicle concentration, infrastructure, urban sprawl and decreased over time spatial clustering, possibly due to improving underreported data from the overlooked districts. Other regions besides Nepal have also been studied for the same zone clustering and spatial concentration trends. These have been captured in Thailand [33], Turkey [34], Hungary [35], Germany [36], Nigeria [37], Ethiopia [38] and Jordan [39]. In these densely populated urban areas, the incidence of road traffic accidents increases. A combination of these features is provided by the Nepali context as well, which includes swift urban expansion, inadequate road safety elements, loose enforcement of traffic regulations, increased vehicle numbers, and motorization.

From fiscal years 2019–2022, the vehicle density and RTAs rates per 100,000 population exhibited and emphasized that the bivariate LISA analysis recorded significant and proven vehicle type correlation to RTAs rate. It is important to recognize that trucks, tippers, buses, cars and motorcycles showed unique spatial patterns indicating high and low severity hot spots of RTAs severity. The HH clusters for all vehicle types and years in the analysis are only formed by Kathmandu, Lalitpur and Bhaktapur districts. These districts are densely populated and resource strained, facing severe traffic congestion and vehicle overcrowding [40]. Their HH cluster stability suggested that with vehicle, infrastructure and traffic control policies of these districts. On the opposite side, remote rural Bajhang, Bajura, Humla and Jumla are the most LL clustered regions. These districts had lower vehicle traffic and are less served by road infrastructure. These areas had consistently low RTAs rates indicating that these remote regions and lower vehicle numbers and slower travel speeds due to rough topography are less likely to encounter road accidents [13]. These regions would, however, benefit from targeted safety enhancements particularly in road traffic accident infrastructure and emergency response frameworks.

The study revealed that heavy vehicles such as trucks and tippers, increased the rate of RTAs in the urban and peri-urban areas where there is notable trade and industrial activity. High-high clusters were common in the areas of Kathmandu, Makawanpur and Dhading where there was a high concentration of commercial vehicles as well as RTAs [41]. This justifies the need for more stringent regulations and safety measures on heavy vehicles as they have a greater likelihood of inflicting severe damage in an accident. For the motorbikes which are the most used means of transport in the country, high-high clusters were noticed in the urban areas with high population density [42]. This highlighted the need as well as targets for keeping safety measures for two-wheeled vehicles alongside with the enforcement of helmet laws and speed regulations. These motorbike clusters of high RTAs, high injury rates and high mortality rates with motorbike users showed how susceptible they were in the traffic accidents. While the bus and car density were spatially similar to other vehicles, their lower Moran’s I value indicated that spatial correlation of RTAs for these vehicles was not as strong as for trucks and motorbikes. Still, in districts such as Kathmandu and Bhaktapur, high rates of RTAs were persistent.

The three years observed with reduction in Moran’s I values demonstrates with most vehicle types, the spatial correlation has weakened. There may still be some districts where vehicle-related accidents are concentrated, but where vehicle-related accidents are occurring in other districts, it has redistributed, likely due to traffic flow, changes in vehicle registrations or the enforcement of local safety regulations. As a whole, the analysis provided an understanding of the spatial distribution of RTAs in Nepal by vehicle type and demonstrated the need for prompt action in identified areas especially in high vehicle use urban areas. Moreover, the analysis indicated that less populated and rural areas, despite being low-risk areas for the time being, need to be factored in for the long-term planning of road safety measures. High risk districts of Nepal could greatly benefit from the enforcement of vehicle safety standards, road construction and traffic control ultimately leading to a reduction in road traffic accidents in Nepal.

The current study has agreed with the results of the Turkey study which performed a spatial analysis with methods such as Moran’s I, LISA, Spatial Lag Model, Spatial Error Model, Spatial Durbin Model and General Nesting Spatial Model to study regional traffic accidents and found that LISA analysis did cluster provincial capitals as a local cluster and the fixed-effect models under various spatial structures showed positive relationships of RTAs with the number of cars, vans, private cars and the lengths of asphalt roads [34]. Thailand study using Global Moran’s I and LISA has shown that the approximately 94.1 percent of RTAs in Khon Kaen Municipality were riders of motorcycles and were not caused by other types of vehicles [43]. These results matched our findings which showed enduring High-High clusters within urban districts including Kathmandu, Lalitpur and Bhaktapur, areas with heavy traffic and extensive two-wheeler utilization but insufficient infrastructure. The appearance of fresh clusters in semi-urban Terai districts including Sarlahi and Sunsari demonstrates that RTAs are expanding their spatial impact across regions outside established urban hubs. Considering the findings, this study has several key policy recommendations. First, specific motorcycle and van enforcement should be prioritized in urban hotspots including stricter policies on licensing, speed enforcement, helmet enforcement and usage monitoring. Second, urban infrastructure modifications such as designated motorcycle lanes, improved crosswalks for pedestrians and traffic calming measures should be added in areas with recurring crash clusters. Third, the primary concentration of vehicle crashes in outer districts calls for centralized roadway risk safety policies, allowing regional and municipal governments to respond to area-specific risk patterns. Fourth, the study shows the practical relevance of spatial models such as Moran’s I and LISA for high-risk zone identification suggesting the tools should be incorporated into routine monitoring frameworks for the Ministry of Physical Infrastructure and Transport and Nepal Police. Finally, the intricacy of RTA clustering impacted by vehicle type, urban concentration and road conditions underscores the need for coordinated attention from multiple sectors: transport, health, urban planning and policing. It is recommended to adopt a new public health approach (health promotion) including health literacy on RTA, provision of good roads and other safe driving facilities and context specific RTA preventive policies and strategies to be implemented in line with the support of international successful health promotion best practices [44,45].

### Strengths and Limitations

This study provides several strengths for further policy development. First, its nationwide coverage of all 77 districts across diverse geographic and socio-economic conditions enhances the statistical power and generalizability of the findings. Second, the comprehensive spatial analysis can facilitate precise identification of high-risk areas and contributing factors, providing a strong evidence base for targeted policy interventions. Third, the visual and descriptive presentation of spatial patterns through mapping techniques improves interpretability, increasing the study’s utility for both researchers and policymakers.

However, several limitations should be acknowledged. Firstly, reliance on secondary data based on police reports, may introduce potential biases, including underreporting of minor accidents or misclassification of vehicle types. Secondly, the lack of specific rural-urban differentiation represents a missed opportunity to examine how settlement patterns influence accident risk, an important consideration for future study. Thirdly, the study period (FY 2019−2022) coincided with COVID-19 travel restrictions in Nepal, which may have intentionally depressed accident rates and misrepresented typical traffic patterns. These pandemic-related effects should be considered when interpreting the findings and comparing them to non-pandemic periods.

## Conclusion

This study reveals significant spatial clustering of road traffic accidents across Nepal, with urban centers particularly in Kathmandu, Lalitpur and Bhaktapur as emerging as persistent high-risk zones for all vehicle types including trucks, tippers, motorbikes, buses and cars. These areas face aggravated risks due to dense traffic, inadequate infrastructure and mixed-vehicle flows. The findings explored that the need for multi-systematic interventions by making traffic enforcement laws strictly and public awareness campaigns in urban centers, developing alongside infrastructure improvements in semi-urban and rural areas. In relation to RTAs, a comprehensive approach integrating engineering upgrades with behavioral interventions and policy reforms, including Health in All Policies, is needed. Future studies should focus on assessing the effectiveness of these interventions, monitoring changes in risk behavior, and supporting evidence-based strategies for road safety.
